# Supplementary material for: Characterization of GEXP15 as a Potential Regulator of Protein Phosphatase 1 in Plasmodium falciparum
Source: Int J Mol Sci. 2023 Aug 10;24(16):12647. doi: 10.3390/ijms241612647 (PMC10454571; doi:10.3390/ijms241612647)
Supplement: Supplementary file 1 [file ijms-24-12647-s001.zip › ijms-2515600-supplementary/Supplementary material/IJMS supplementary material proof reading-R2.docx]

Supplementary Material

**Characterization of GEXP15 as a potential regulator of Protein Phosphatase 1 and partner of ribosomal complex in Plasmodium falciparum.**

**Hala Mansour ^1^, Alejandro Cabezas-Cruz ^2^, Véronique Peucelle^1^, Amaury Farce^3^, Sophie Salomé-Desnoulez^4^, Ines Metatla^5^, Ida Chiara Guerrera^5^, Thomas Hollin^1,6^*, Jamal Khalife^1^***

^1^ Center for Infection and Immunity of Lille, Biology of Apicomplexan Parasites, UMR 9017 CNRS, U1019 INSERM, University of Lille, Institut Pasteur de Lille, Lille, France.

^2^ ANSES, INRAE, Ecole Nationale Vétérinaire d’Alfort, UMR BIPAR, Laboratoire de Santé Animale, Maisons-Alfort, France.

^3^ University of Lille, Inserm, CHU Lille, U1286 - Infinite - Institute for Translational Research in Inflammation, F-59000 Lille, France.

^4^ University of Lille, CNRS, Inserm, CHU Lille, Institut Pasteur de Lille, US 41 - UAR 2014 - PLBS, F-59000 Lille, France

^5^ Proteomics platform 3P5-Necker, Université Paris Descartes - Structure Fédérative de Recherche Necker, INSERM US24/CNRS UMS3633, Paris, France

^6^ Department of Molecular, Cell and Systems Biology, University of California Riverside, Riverside, CA, USA.

*** Correspondence:**Corresponding Author
jamal.[Khalife@pasteur-lille.fr](mailto:Khalife@pasteur-lille.fr)
thollin@ucr.edu

# Supplementary Figures


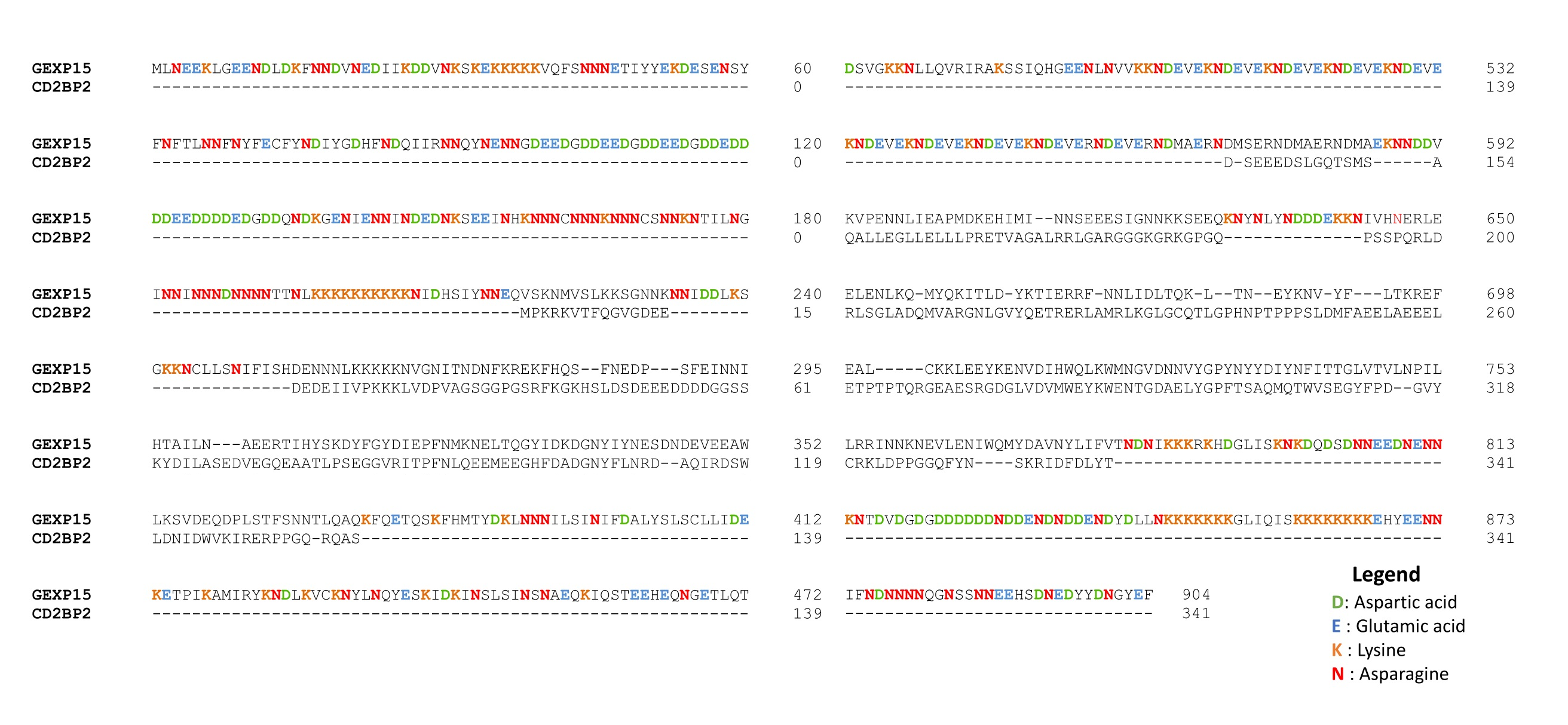


**Supplementary Figure S1. The protein sequence alignment of PfGEXP15 and HsCD2BP2.** The alignment was performed using ClustalW. Colored amino acids represent low complexity regions.

#
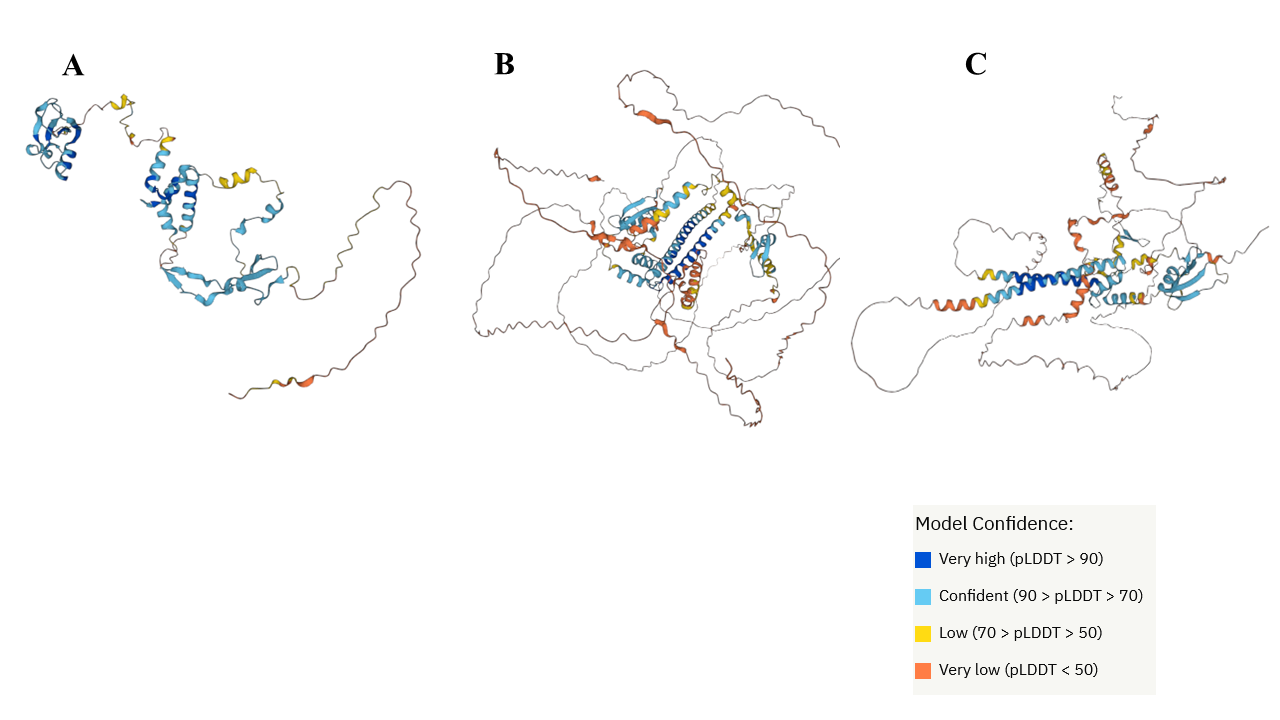


**Supplementary Figure S2. The 3D structure prediction of HsCD2BP2, PfGEXP15 and PbGEXP15.** The models of HsCD2BP2 (A), PfGEXP15 (B) and PbGEXP15 (C) were generated by AlphaFold.


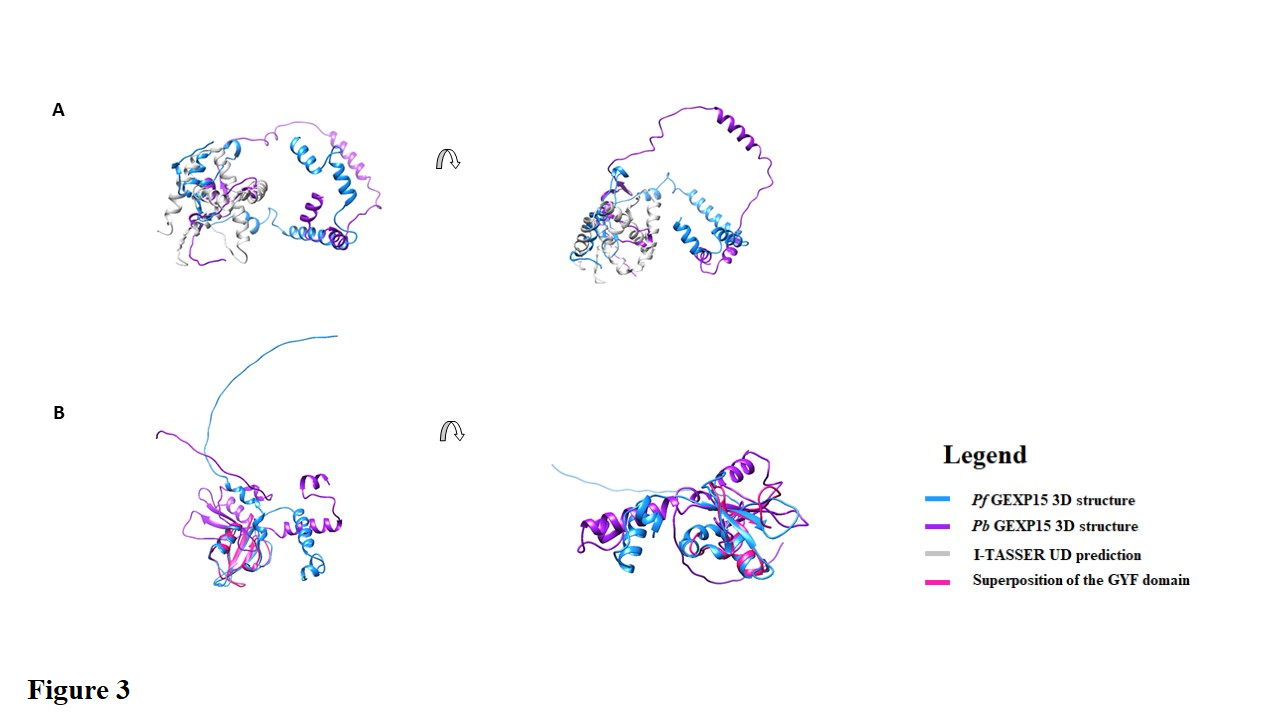


**Supplementary Figure S3. 3D structure prediction of Pf and Pb GEXP15. (A)** The predicted 3D structures of UD and **(B)** GYF domains for PfGEXP15 and PbGEXP15 were retrieved from AlphaFold and are shown in blue and purple, respectively. The protein structure predictions are shown in grey. Both models were superimposed onto the GYF domain NMR structure of human CD2BP2 (PDB entry: 1gyf) using the MatchMaker tool from Chimera (version 1.14).


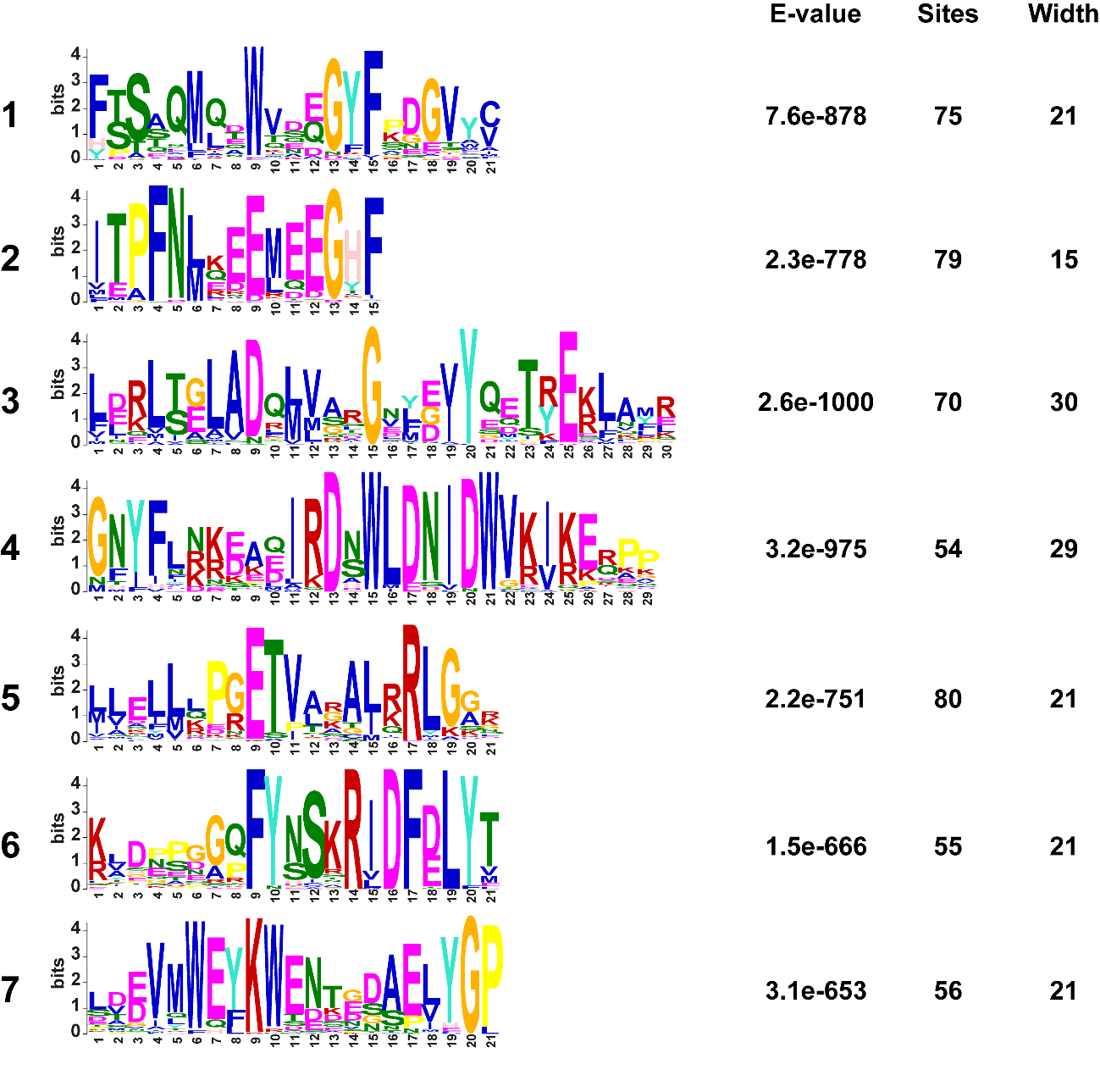


**Supplementary Figure S4. MEME motif search of CD2BP2 homologs in eukaryotes.** The 7 most significant sequence logos identified by MEME are represented, as well as their respective E-value, number of sites and width across the protein sequences. The height and size of the letters represent the amino acid frequency.


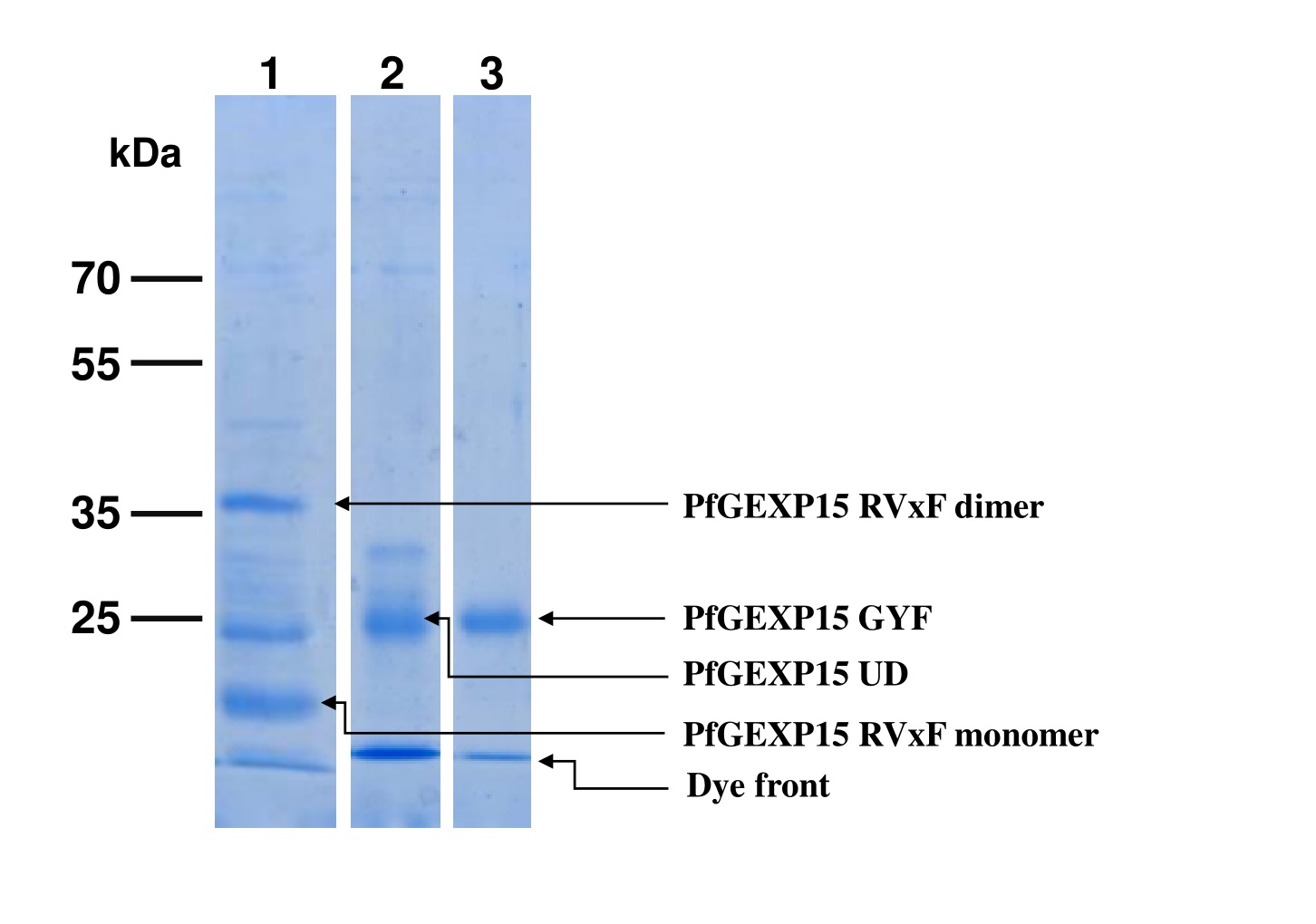


**Supplementary Figure S5.** **PfGEXP15 recombinant proteins.** Coomassie staining of SDS-PAGE of recombinant PfGEXP15 RVxF (lane 1), UD (lane 2), and GYF (lane 3)

**
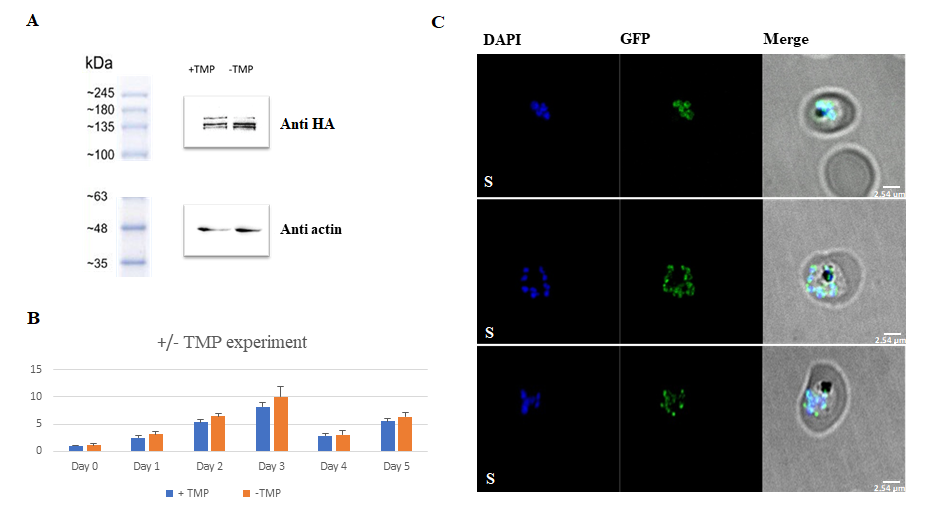
**

**Supplementary Figure S6. TMP removal does not affect parasite growth and PfGEXP15 localization A.** Western Blot analysis representing the total protein extract from a highly enriched cultures of transgenic iKd PfGEXP15 late trophozoites with TMP in lane 1 and without TMP for 12 days (corresponding to around 7 cycles) in lane 2. They were revealed with mAb anti-HA. In the lower panel, anti-actin was used as a positive loading control. 40 million parasites were used in each lane. **B.** Parasitemia of iKd PfGEXP15 line was measured with and without TMP cultures. The results are shown as the mean parasitemia ± SD. (n=4). **C.** Live microscopy showing GFP expressing parasites in transfected cultures without TMP. Parasite’s nuclei were stained with DAPI and transgenic parasites are expressing PfGEXP15-GFP-DDD-HA. Merged images showed the protein colocalization.


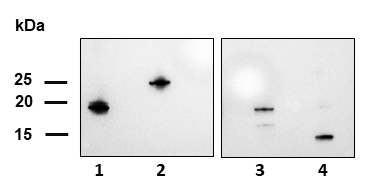


**Supplementary Figure S7.** Western blot analysis representing RVxF motif (lane 1), tetR protein (lane 2), UD (lane 3) and GYF domain (lane 4) recombinant proteins eluted from nickel beads and detected using anti-His antibodies. The figure was set up from the same western blot at different exposures.


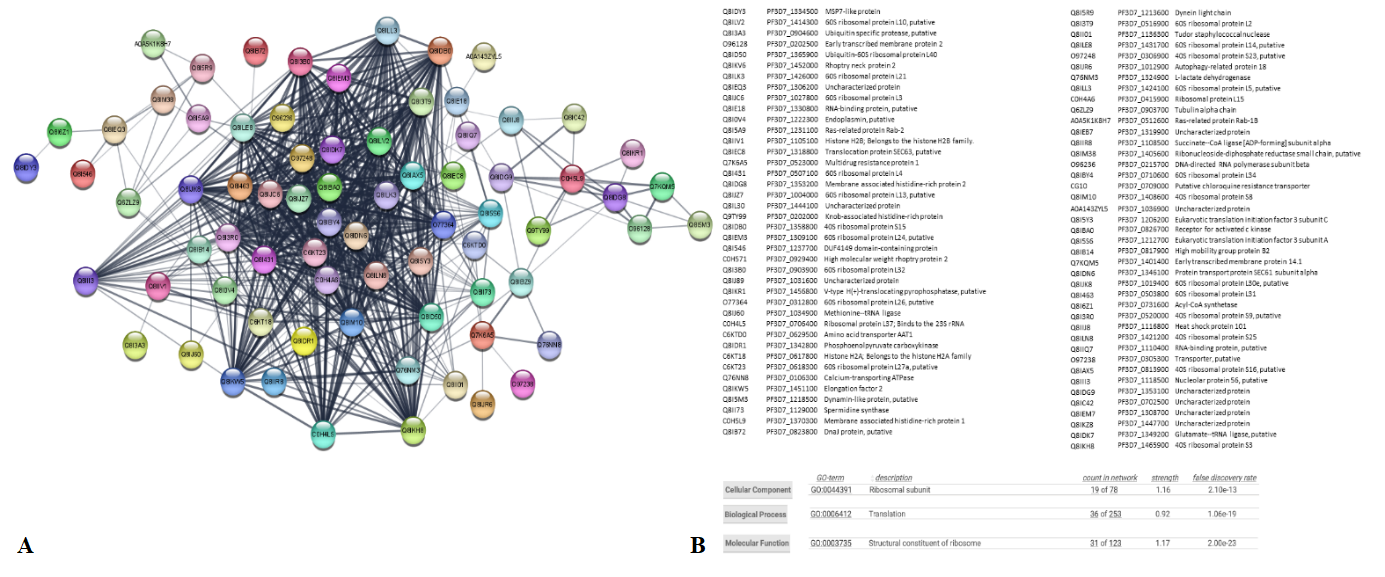


**Supplementary Figure S8. GYF-domain containing protein pulldown analysis.** **A.** STRING network visualization of GYF-interacting proteins using Cytoscape software **B.** List of GYF interacting partners, as well as the processes they may be involved.

# Supplementary Datasheets

# Supplementary Tables
